# Medullary Thyroid Carcinoma: An Update on Imaging

**DOI:** 10.1155/2019/1893047

**Published:** 2019-07-07

**Authors:** Sergiy V. Kushchayev, Yevgeniya S. Kushchayeva, Sri Harsha Tella, Tetiana Glushko, Karel Pacak, Oleg M. Teytelboym

**Affiliations:** ^1^Moffitt Cancer Center, Department of Radiology, 12902 USF Magnolia Drive, Tampa, FL 33612, USA; ^2^Johns Hopkins Hospital, Department of Radiology, 601 N Caroline St, Baltimore, MD 21287, USA; ^3^Diabetes, Endocrinology, and Obesity Branch, National Institute of Diabetes and Digestive and Kidney Diseases, National Institutes of Health, Bethesda 10, Center Dr, Bethesda, MD 20892, USA; ^4^Department of Medicine, Division of Endocrinology, Diabetes and Metabolism, University of South Carolina School of Medicine, 6311 Garners Ferry Rd, Columbia, SC, USA; ^5^Department of Radiology, Mercy Catholic Medical Center, 1500 Lansdowne Ave, Darby, PA 19023, USA; ^6^Eunice Kennedy Shriver National Institute of Child Health & Human Development (NICHD/NIH), 10 Center Dr, Bethesda, MD 20892, USA

## Abstract

Medullary thyroid carcinoma (MTC), arising from the parafollicular C cells of the thyroid, accounts for 1–2% of thyroid cancers. MTC is frequently aggressive and metastasizes to cervical and mediastinal lymph nodes, lungs, liver, and bones. Although a number of new imaging modalities for directing the management of oncologic patients evolved over the last two decades, the clinical application of these novel techniques is limited in MTC. In this article, we review the biology and molecular aspects of MTC as an important background for the use of current imaging modalities and approaches for this tumor. We discuss the modern and currently available imaging techniques—advanced magnetic resonance imaging (MRI)-based techniques such as whole-body MRI, dynamic contrast-enhanced (DCE) technique, diffusion-weighted imaging (DWI), positron emission tomography/computed tomography (PET/CT) with 18F-FDOPA and 18F-FDG, and integrated positron emission tomography/magnetic resonance (PET/MR) hybrid imaging—for primary as well as metastatic MTC tumor, including its metastatic spread to lymph nodes and the most common sites of distant metastases: lungs, liver, and bones.

## 1. Introduction

Thyroid neuroendocrine cells were first described in 1876 by Baber, and they were named C cells (CC) due to the secretion of calcitonin (CTN) by Pearse in 1966 [1]. The human thyroid gland contains less than 0.01–0.1% CTN-producing parafollicular C cells. Medullary thyroid carcinoma (MTC) arises from these cells and accounts for 1–2% of thyroid cancers. Although the majority of MTCs are sporadic, 25% of cases are hereditary and are found in multiple endocrine neoplasia (MEN) 2A or 2B syndromes, or as part of familial MTC based on a specific germline mutation in the* RET *proto-oncogene [2].

MTC is frequently aggressive; on initial presentation only about half of patients (48%) have localized disease, while 35% have a tumor extending beyond the thyroid into the surrounding tissues or regional lymph node metastases, and 13% have distant metastases typically to the lung, liver, or bones [3, 4]. There is still no single optimal diagnostic imaging method to reveal all MTC recurrences or metastases, and therefore concurrent use of several imaging modalities is often required to provide the information needed for directing the management.

New functional techniques—such as dynamic contrast-enhanced (DCE) magnetic resonance imaging (MRI), diffusion-weighted imaging (DWI) MRI, and positron emission tomography/computed tomography (PET/CT) using 18-fluorodeoxyglucose (18F-FDG), fluorine-18-dihydroxyphenylalanine (18F-FDOPA), and somatostatin (SST) analogs labeled with 68Ga—have been recently introduced for the evaluation of oncologic patients. However, clinical applications of these modalities in MTC are very limited. The main aim of this article is to summarize current and outline new potentially helpful radiologic techniques, which have not yet been tested for MTC, but were found to be beneficial for other (neuroendocrine) malignancies.

## 2. Modern Imaging Modalities and Techniques

### 2.1. Ultrasound

Ultrasound (US) remains the most commonly used and preferred imaging modality for the evaluation of the location and characteristics of neck masses and thyroid nodules. US can be helpful in the evaluation of regional nodal metastases in patients with proven MTC, assessment for suspected recurrent disease, and high-risk patients for occult MTC. Color and Power Doppler techniques are useful for the evaluation of the vascular pattern of cervical lymph nodes. Fine needle aspiration with assaying CTN in the washout fluid helps diagnose MTC preoperatively [5].

### 2.2. Computed Tomography

The primary use of CT in MTC is to detect and characterize cervical and mediastinal lymph nodes and evaluate lung parenchyma for metastases. For patients with suspected hepatic metastases, multiphase CT scanning includes nonenhanced, late arterial phase, portal venous phase, and delayed phase imaging; however, it is less effective than MRI techniques. Nonenhanced CT provides characterization of bony changes secondary to metastases; however, the evaluation of the bone marrow infiltration, spinal canal, and soft tissue is limited and requires further imaging with MRI.

### 2.3. Magnetic Resonance Imaging

**Figure 1:**
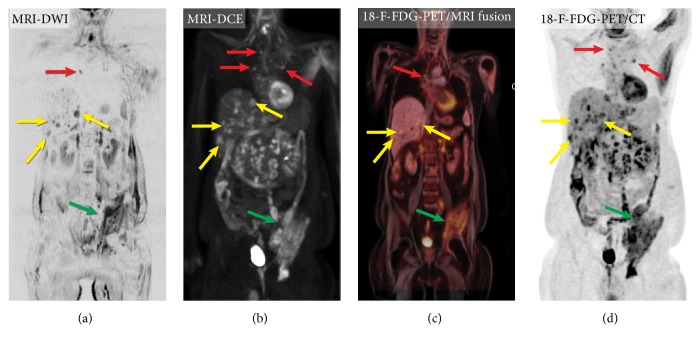
Imaging of metastatic medullary thyroid carcinoma using different radiological techniques: (a) whole-body magnetic resonance imaging, diffusion-weighted sequence (DWI), (b) whole-body contrast-enhanced magnetic resonance imaging, T1 weighted images (3D rendering DCE); (c) positron emission tomography/magnetic resonance imaging (18F-FDG-PET/MR imaging fusion technique); (d) positron emission tomography/computed tomography with 18F-FDG (18F-FDG PET/CT). Please note the excellent visualization of the multiple liver metastases (yellow arrow), mediastinal metastases (red arrow), and a large left iliac bone metastasis (green arrows) on DWI and DCE, comparable with 18F-FDG PET/CT, although they interrogate completely different biophysical tissue properties. Note that the images are not quite coregistered (arms up during PET acquisition and with arms down during MR imaging).

MRI provides excellent visualization of metastatic lesions in soft tissues, intra-abdominal lymph nodes, bones, and liver and can help evaluate the extent of the disease during the initial staging and assess the treatment response. Several new MRI techniques in oncologic imaging—including whole-body MRI (WB-MRI), DCE technique, DWI, and integrated PET/MR hybrid imaging—not only allow the evaluation of the biological and functional effects of treatment, but also offer the opportunity to evaluate treatment response, study tumor pathophysiology, and heterogeneity and may also predict clinical outcomes, particularly in the setting of novel adjuvant therapies [6] (Figure 1).

*WB-MRI* is a potentially useful technique for the initial staging and evaluation of treatment response. The technique is particularly useful in children and young adults because it does not use ionizing radiation [7].

*DWI *measures the diffusion of water molecules (Brownian movement) within a given tissue, which is mainly dependent on cellular density. Tissues with a high cellularity—including malignancies—have a smaller extracellular space, resulting in restricted water molecule diffusion and a high DWI signal. The degree of proton diffusion restriction can be quantified via the apparent diffusion coefficient (ADC) [8]. Numerous studies have shown that ADC values in a number of nonthyroid tumors can be used to access treatment response [9]. WB scanning utilizing DWI sequence (WB-DWI) has recently been introduced as an effective evaluation of metastases burden in oncology (Figure 1(a)). WB-DWI is not a replacement for PET/CT for the evaluation of oncological patients; indeed, these techniques are complementary because they interrogate completely different biophysical tissue properties (glucose metabolism vs. cellular density) [10]. WB-DWI might be of value in recognizing non-FDG–avid metastases and evaluating anatomic regions with high physiologic radiotracer uptake. However, at this point there are no studies that have evaluated the utility of DWI techniques for patients with metastatic MTC.

*DCE-MRI* is being used in oncology to measure properties of tumor microvascular structure and permeability. DCE-MRI becomes an attractive modality for evaluating antiangiogenic cancer therapies, assessing changes in tumor vasculature, and predicting tumor shrinkage [11]. This technique may have a clinical application in MTC, as targeting angiogenesis produced the most impressive clinical treatment responses for metastatic MTC to date [12]. Only one study reported the exploratory use of DCE-MRI in 10 patients in order to evaluate tumor perfusion as a surrogate of the antiangiogenic activity of sorafenib [13]. Since capillary perfusion determines the delivery of drugs to tumor cells, recent studies have used the* in vivo* measurement of capillary perfusion by DCE-MRI to monitor perfusion changes in response to antiangiogenic agents [8] (Figure 1(b)).

*PET/MR hybrid imaging* is an innovative technique still under investigation. MR imaging can depict metastatic bone marrow involvement before the development of structural damage that is visible on a CT scan. A large meta-analysis based on 57 publications showed that the diagnostic performance of simultaneous PET/MR imaging was similar or even better than PET/CT for most anatomical locations and most oncological diseases; however, PET/CT was superior for small lung nodule detection [14] (Figure 1(c)).

### 2.4. Nuclear Medicine Techniques

**Figure 2:**
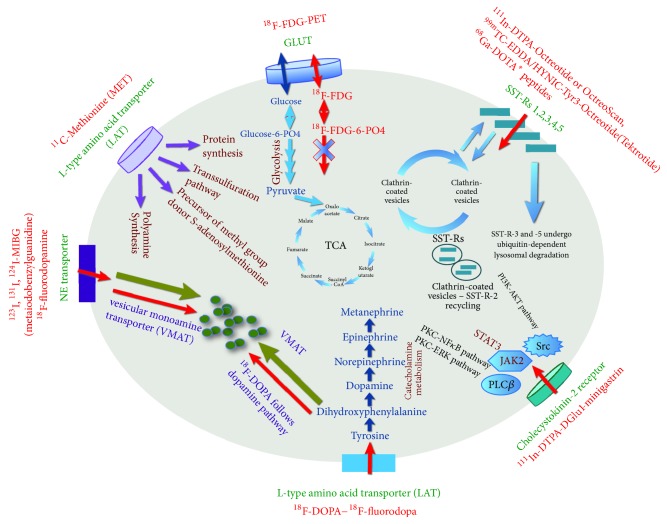
Mechanisms of uptake and localization of different radiopharmaceuticals, which can be used for medullary thyroid carcinoma. Please see explanations in the text.

There are numerous conventional (such as a bone scan or metaiodobenzylguanidine (MIBG) scintigraphy) and advanced (PET/CT scans with different radiopharmaceuticals) nuclear medicine techniques that can be useful for patients with MTC. There is no general agreement on their routine use in MTC [15, 16] (Figure 2).

Several studies have evaluated the diagnostic accuracy of different radiotracers for PET/CT in patients with suspected recurrent MTC. The majority of these studies were limited by the small number of patients, as the disease is rare. Some of the studies showed conflicting results. In 2012 Treglia et al. published two large meta-analyses evaluating the diagnostic accuracy of PET/ CT with 18F-FDOPA and 18F-FDG [17, 18]

#### 2.4.1. 18F-FDOPA

18F-FDOPA PET/CT represents the single best modality for whole-body MTC metastasis detection [19]. 18F-FDOPA PET/CT is particularly suitable for the detection of small metastatic lymph nodes (around 6 mm) [20]. Moreover, 18F-FDOPA PET/CT is a sensitive technique to identify liver metastases not detected by 18F-FDG as well as MTC metastases in unusual locations [20]. In patients with rising tumor markers, detection rates are considerably higher than with other imaging methods (66 % patient-based, 71 % lesion based), which is of clinical significance since imaging with 18F-FDOPA is often performed in patients who have negative results on conventional imaging and/or 18F-FDG PET/CT [18]. Using the proposed CTN cutoff for additional imaging according to the American Thyroid Association (ATA) guidelines [15], the sensitivity of 18F-FDOPA PET for metastatic/recurrent MTC is 79-100% [19, 21]. In the meta-analysis by Treglia et al., the sensitivity of 18F-FDOPA PET or PET/CT for metastatic/recurrent MTC was 73% if CTN values were above 150 pg/ml (551 pmol/l) [18].

18F-FDOPA PET is superior to 18F-FDG PET in the evaluation of metastatic/recurrent MTC, with a higher patient-based sensitivity (64% vs. 48%, respectively; range, 38–83% vs. 17–64%) and lesion-based sensitivity (72% vs. 52%, respectively; range, 52–94% vs. 28–62%) [22]. The sensitivity increases when both imaging modalities are used [22]. The patient-based detection rate increases significantly with CTN levels >150 pg/ml (551 pmol/l) vs. CTN<150 pg/ml (551 pmol/l) for both 18F-FDOPA PET/CT (sensitivity 91% vs. 29%) and 18F-FDG PET/CT (sensitivity 73% vs. 14%) [20]. The diagnostic accuracy of 18F-FDOPA and 18F-FDG PET/CT increases significantly when a carcinoembryonic antigen (CEA) cutoff of > 5 ng/mL is used (sensitivity of 81% and 73%, respectively, vs. 43% for 18F-FDOPA PET/CT and 14% for 18F-FDG PET/CT when CEA < 5 ng/mL) [20]. In a large meta-analysis, Treglia et al. also found higher detection rates of 18F-FDOPA PET or PET/CT when setting the cutoff of CEA at 5 ng/mL (detection rate of 64% with CEA > 5 ng/mL vs. 48% with CEA ≤ 5 ng/mL) [18].

#### 2.4.2. 18F-FDG PET/CT

18F-FDG accumulates in neoplastic cells using glucose as an energy source mainly according to their proliferative activity. However, neuroendocrine tumors (NETs), including MTC, frequently show an indolent course and, consequently, a low 18F-FDG uptake [23]. A detection rate of 18F-FDG PET/CT on a per patient-based analysis ranged from 24 to 95% with a pooled estimate of 59% (95% CI: 54–63%) and improved in patients with increased serum CTN and CEA levels, and shorter serum CTN and CEA doubling times [4, 17, 18, 24]. The lowest CTN levels with positive 18F-FDG PET have a wide range in the literature from 129 to 816 pg/ml (473–2996 pmol/l) [25–27]. 18F-FDG PET rarely detects disease in patients with CTN levels below 500 pg/ml (1836 pmol/l) [26]. Sensitivity was 78% and 20% for CTN above 1000 (3671 pmol/l) and below 1000 pg/ml (3671 pmol/l), respectively [26]. The average CTN level in patients with a positive 18F-FDG PET was found to be 2311 pg/ml (8483 pmol/l) with a range of 51–247,000 pg/ml (187–906737 pmol/l) in one study [27] and 7260 pg/mL (26651 pmol/l) with a range of 106–541,000 pg/ml (389–198,6011 pmol/l) in another [26]. However, a negative 18F-FDG PET has been described even with a CTN of 55,200 pg/ml (202,639 pmol/l) [26]. The maximum standardized uptake value (SUV max) has been shown to correlate with CTN levels [24, 28]. 18F-FDG PET/CT should not be considered as first-line diagnostic imaging methods in patients with suspected recurrent MTC but could be very helpful in detecting recurrence in those patients in whom a more aggressive disease is suspected [4, 29].

#### 2.4.3. Somatostatin Receptor Scintigraphy

Somatostatin (SST) is a regulatory peptide widely distributed in the human body that binds to somatostatin receptors (SST-R types 1–5). SST-R expression can be visualized using conventional scintigraphy or PET/CT with a number of different tracers. 111In-octreotide has high affinity for SST-R-2, but poor nuclear imaging properties with detection rates between 20 and 64% [30]. Newer radiolabeled SST-R analogs have been developed. Using 1,4,7,10-tetraazacyclododecane-1,4,7,10-tetraacetic acid (DOTA) as a universal chelator that provides stable complexes with 111In, 68Ga, 177Lu, and 90Y and is used to label SST-R analogs maintains biological activity that is sufficient for imaging and therapy. 68Ga-DOTA, 68Ga-DOTANOC (SST-R-2,3,5), 68Ga-DOTATATE (SST-R-2), 68Ga-DOTATOC (SST-R-2 and 5), 68Ga-DOTA-NOC (SST-R-2,3,4 and 5), and 99mTc-EDDA/HYNIC-Tyr3-Octreotide (Tektrotide) have important clinical implications. PET/CT with 68Ga–labeled SST-R compounds is a valuable diagnostic tool for patients with NETs. Experience in metastatic and recurrent MTC is limited compared to 18F-FDG and 18F-DOPA PET/CT with inferior results compared with the other two tracers as MTC has lower density and an inhomogeneous expression of SST-R compared to other NETs [31]. 68Ga-DOTATATE PET/CT is superior to 111In-octreotide SPECT/CT for the detection of recurrent MTC demonstrating a significantly higher number of lesions [31]. As is the case with 18F-FDG and 18F-FDOPA PET/CT, the detection rate of SST-R analogs PET/CT in recurrent MTC increases in patients with higher serum CTN levels [23]. Nevertheless, SST-R analogs PET/CT may have an additional role, as this method could be useful in the selection of patients with inoperable tumors for peptide receptor radionuclide therapy with 90Y-DOTA0-Tyr3-octreotide (90Y-DOTATOC) or 177Lu-DOTA0-Tyr3-octreotate (177Lu-DOTA-TATE) [30].

A meta-analysis of nine studies devoted to the diagnostic performance of SST-R analogs PET or PET/CT (including 68Ga-DOTATATE, 68Ga-DOTANOC, 68Ga-DOTANOC, and 68Ga-DOTALAN) in patients with recurrent MTC demonstrated a suboptimal detection rate of 64% (a per patient-based analysis), which increases to 83% for CTN >500 ng/L [32]. Overall, the diagnostic performance of SST-R analogs PET or PET/CT in recurrent MTC is overall lower compared to 18F-FDOPA PET/CT [32]. Although 68Ga-DOTATATE PET/CT is not an optimal whole-body imaging technique as a single imaging modality in patients with MTC, it detects 100% for bone metastases and it was found to be superior to bone scan that identified 44% of osseous lesions [33].

#### 2.4.4. Cholecystokinin Receptor Subtype 2 (CCK2R)/Gastrin Receptors and Gastrin Receptor Scintigraphy

The cholecystokinin receptor subtype 2 is an important promoter of tumor growth. It has been shown to be expressed in normal C cells and overexpressed in numerous NETs, including MTC [34, 35]. Blaker et al., based on an analysis of 19 MTC patients, found that CCK2Rs could be detected in almost all early-stage tumors, such as T1 and T2 stages, while further growth and potential loss of cell differentiation, i.e., in T3 and T4 primary and metastatic tumors, might be associated with a loss of CCK2Rs [35]. Two DOTA-minigastrin analogs radiolabeled with 111In and 68Ga were evaluated in a preclinical* in vivo* model and showed higher uptake than 68Ga–labeled cyclic DOTA-peptides [36]. The injection of radiolabeled minigastrin was associated with mild side effects such as nausea, flushing, and hypotension that are similar to pentagastrin testing [37]. Since there is no physiologic uptake in liver and spleen, 111In-DTPA-DGlu1-minigastrin favors the detection of metastatic lesions in these organs [38]. The combination of CT with a 111In-DTPA-DGlu1-mini-gastric scan resulted in the detection of 96.7% of known lesions [37]. However, it was not accurate for occult disease [37]. The main limitations of this technique are the limited availability and that single-photon emission computed tomography (SPECT-CT) scan does not provide sufficient spatial resolution for the precise detection of lesions and high-precision surgical planning.

#### 2.4.5. MIBG

Both 131I-MIBG and 123I-MIBG are available for diagnostic purposes. With a cumulative sensitivity for MTC between 30 and 50% [39, 40], they are not recommended except for imaging of pheochromocytomas in case of MEN2, a syndrome where MTC is found.

#### 2.4.6. 11C-Methionine

Methionine is an essential amino acid necessary for protein synthesis. However, this tracer has a considerable nonprotein metabolism and generates substantial amounts of nonprotein metabolites, making the correct quantification of protein synthesis difficult [41]. 11C-Methionine was more sensitive to the detection of cervical lymph nodes when compared to 18F-FDG, but not better than neck US or a combination of 18F-FDG PET with neck US for lymph node detection [25]. 11C-Methionine, as well as 18F-FDG, was more sensitive in cases of elevated CTN with a cutoff > 370 pg/ml (1358 pmol/l); however, the high physiologic uptake of 11C-MET in the liver that limits visualization of of hepatic metastases [25].

#### 2.4.7. Immunoimaging

Using directly labeled antibodies, their fragments, or antibody-derived recombinant constructs has been proposed for the radionuclide targeting of tumors. Immuno-PET using anti-CEA antibodies labeled with 111In, 131I, or 68Ga was reported to be potentially accurate for detecting relapsing MTC [42]. Further studies are required to confirm the accuracy of this new technique [42].

#### 2.4.8. Bone Scan

Although, the current 2015 ATA guidelines recommend bone scintigraphy for patients with extensive neck disease, regional or distant metastases, and a serum CTN level greater than 500 pg/ml (1836 pmol/l), the diagnostic value of this technique for MTC is not well-investigated. As patients with suspected distant metastases due to MTC, including bone metastases, should undergo PET/CT or PET/MRI with 18F-FDG and 18F-FDOPA anyway, these techniques appear more efficient for the detection of bone lesions, rather than bone scintigraphy.

## 3. Clinical Application of the Imaging Techniques

### 3.1. Preoperative Evaluation

**Figure 3:**
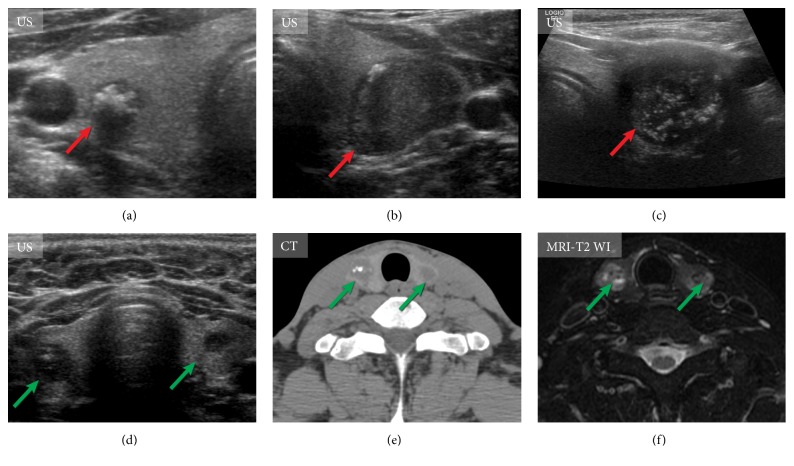
Imaging of the primary tumor in medullary thyroid carcinoma. (a)-(c) Ultrasound (US) features of primary medullary thyroid carcinoma of different patients (red arrows). US of the thyroid gland shows markedly hypoechoic nodules with marked calcifications. (d)-(f) US of the neck, computed tomography, and magnetic resonance tomography (T2 weighted images of the neck) of the same patient with bilateral medullary thyroid carcinomas (green arrows).

Most patients with sporadic MTC have unifocal disease and present with a palpable neck mass usually in the fifth or sixth decade of life. These tumors tend to be located in the posterior thyroid and therefore can compress or invade local structures, causing hoarseness, dysphagia, or respiratory difficulty [43]. Sonographic findings of primary MTC are often not specific. A large meta-analysis that included 157 malignancies showed that the majority (83%) of tumors were hypoechoic, 38% had irregular margins, 36% demonstrated microcalcifications, and 27% showed macrocalcifications (Figure 3). Compared with papillary thyroid carcinoma, MTCs were found to be larger, and more frequently showed cystic changes and homogeneous echotexture of the solid portion [44]. The US appearance of MTC nodules was divided into two categories: m-MTC type with aggressive US features and b-MTC type without aggressive US features [45, 46]. Metastatic spreading of the malignancy to lymph nodes, extrathyroidal invasion, more-advanced TNM stage, and higher postoperative CTN levels were more frequent in the m-MTC group [45, 46]. CT and MRI are generally used for the evaluation of larger thyroid nodules (greater than 3 cm in diameter) and to evaluate substernal extension and invasion into adjacent structures [47]. Sonographic patterns proposed by the 2015 ATA Thyroid Nodules and Differentiated Thyroid Cancer guidelines were primarily aimed at differentiated thyroid cancer, but these recommendations were also found to perform well in MTC [48].

Cytology evaluation alone is not enough for preoperative evaluation and to guide initial surgery [49]. Ultrasound-guide fine needle aspiration cytology (FNAC) of a thyroid nodule cannot always reliably distinguish between MTC and other thyroid neoplasms including adenomas. Sensitivity of FNAC was shown to be 63% vs. 98% for serum CTN measurement with only 74.5% cases diagnosed by FNAC in patients with elevated CTN level [50]. FNAC is a very important measure in the preoperative workup of patients with thyroid nodules; however, it is controversial due to questions of efficacy, accuracy, and cost-effectiveness [51]. Serum CTN has also some limitations including high false positive results, low positive predictive value, and lack of agreement for CTN threshold to suspect MTC [51, 52]. Diagnosis of MTC in thyroid nodules with undetermined cytology using MTC gene classifier was shown to demonstrate a high sensitivity of 97.9%, specificity of 99.8%, and positive and negative predictive values of 97.9% and 99.8%, respectively [51].

If the preoperative diagnosis of MTC is missed and surgery starts with a diagnostic hemithyroidectomy, reoperation is needed to perform total thyroidectomy. A definitive diagnosis is often made only on surgical histopathology [53].

As surgery remains the only curative treatment for patients with MTC, the ultimate goal of preoperative evaluation is to identify all distant metastatic lesions and, most importantly, metastatic lymph nodes. Lymph node involvement is seen in 35–50% and distant metastases are present in 10–15% of patients at the time of diagnosis [2]. Different imagining modalities show distinct detection rates for lesions in different organs; therefore, radiological workup should be tailored based on the most suitable method for each organ (system).

**Figure 4:**
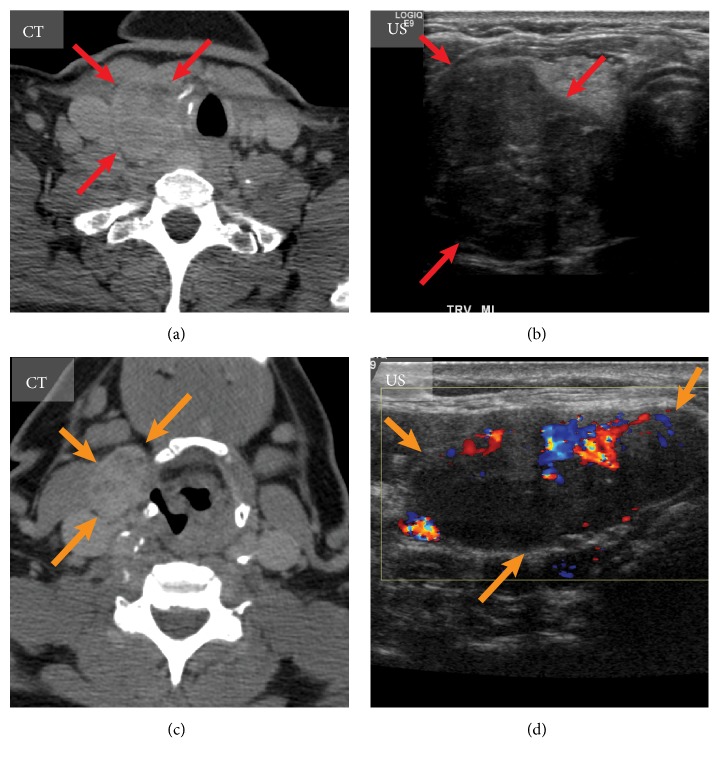
Imaging of the medullary thyroid carcinoma with metastasis to lymph node. Medullary thyroid carcinoma of the right thyroid lobe (red arrows) visualized on axial computed tomography (CT) (a) and transverse ultrasound (US) scan (b). A large metastatic lymph node (orange arrows) is demonstrated on axial CT of the neck (c) and sagittal US (d).

Preoperative evaluation of the extent of the primary tumor and lymph node involvement with US is an essential procedure. In cases when the primary tumor is 1 cm or larger, the risk of lateral neck metastasis is higher, and careful US examination of the lateral neck nodes should be performed [54] (Figure 4). However, 36% of patients might have false negative results on preoperative neck US, especially in central neck compartment [55]. Thirty-eight percent of patients with node-negative MTC were shown to fail normalization of CNT level after surgery despite extensive surgical treatment, which is suggestive of the possibility of distant metastases [56]. More intensive imaging, invasive diagnostic techniques, and extension of follow-up can likely improve the ascertainment rates of distant metastases [56]. Aggressive evaluation of the suspicious appearing neck lymph nodes should undergo FNAC with CTN assay of washout fluid. The current ATA guidelines recommend imaging procedures to exclude metastatic MTC for patients with extensive neck disease and signs/symptoms of regional or distant metastases and all patients with CTN > 500 pg/ml (1836 pmol/l) [15].

This recommendations is based on the study published by Machens and Dralle in 2010 [57]. Patients who underwent thyroidectomy and lymph node dissection and had preoperative CTN <100 pg/ml (367 pmol/l) could be biochemically cured [57]. However, only 81% of patients with a CTN level of 100–500 pg/ml (367–1836 pmol/l) who underwent surgery had a normal postoperative CTN level [57]. Thus, every fifth patient with CTN levels of 100–500 pg/ml (367–1836 pmol/l) probably had metastatic disease, which could not be resected during the surgery or might have been located outside of the surgical area, while imaging findings were not analysed in this study. Moreover, preoperative CTN levels do not always reflect the actual stage of a patient's disease [58, 59]. These levels correlate with the tumor mass and the secretory capacity of the malignant cells and, hence, they do depend on the degree of (de)differentiation [59]. While these aspects require further research, patients with preoperative CTN levels above 100 pg/ml (367 pmol/l) might benifit from comprehansive imaging and, idealy, from 18F-FDOPA-PET/CT. This may also be informative for surgical planning (for example, a patient with mediastinal metastatic lymph nodes may require thoracotomy), plan other therapeutic strategies, and establish a “baseline” to assess response to treatments. The use of PET/CT for accurate initial staging and treatment planning is established in many malignancies [60] and can be potentially applied to MTC.

### 3.2. Postoperative Follow-Up Patients with MTC

Follow-up after surgical therapy for MTC typically starts 2–3 months postoperatively by obtaining new baseline CTN and CEA levels. Patients who have undetectable CTN levels postoperatively can be followed with measurements of serum CTN and CEA initially every 6 months for the first year and then annually [15].

In the elegant study of Pellegrini et al. (2003), the active follow-up of the MTC patients allowed the early diagnosis of the metastatic disease at basal serum CTN levels <150 pg/ml (551pmol/l) with regional and/or distant metastatic lesions [15, 58]. Among five patients with CTN level <150pg/ml at the moment of relapse, two patients had distant metastases, two patients had cervical disease, and one had regional and distant metastases. Of note, the imaging-detected relapses were diagnosed in patients who underwent treatment between 1988 and 1998, and available imaging workup included a yearly neck and abdominal US, chest CT without CT of abdomen, and a bone scintigraphy; no MRI or PET/CT was used [58]. In the cohort of 18 patients with imaging-detected relapses in three patients, postoperative CTN level was undetectable. Authors emphasized that even undetectable pentagastrin-stimulated CTN cannot exclude the risk of recurrence and, thus, MTC patients need a prolonged follow-up [58].

**Figure 5:**
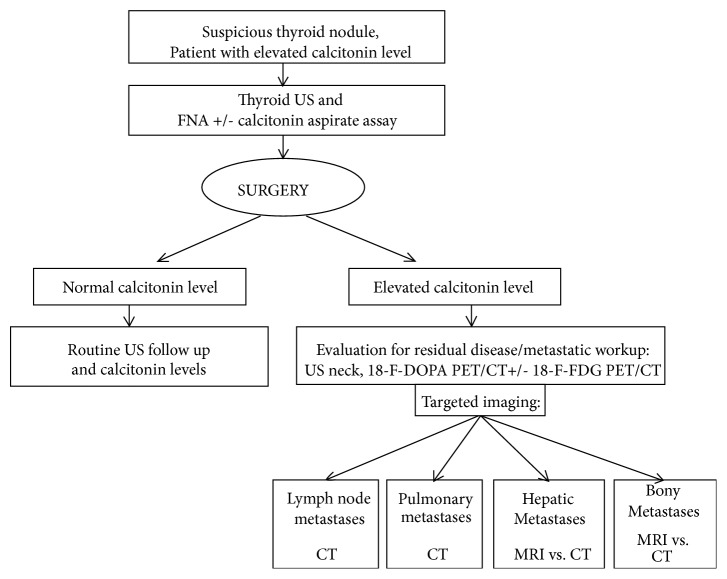
The proposed management approach for patients with medullary thyroid carcinoma. Please see explanations in the text.

MTC includes genetically heterogeneous subtypes of malignancies that demonstrate different clinical courses from very aggressive to slowly progressing. Therefore, an individualized/differentiated approach for imaging and management of patients is optimal. However, currently, there are no meaningful data to offer clinical or histopathological criteria for different imaging protocols. It can be suggested that any patient with elevated postoperative CTN levels should be considered as having metastasis disease of unknown location and should promptly undergo comprehensive imaging: US, CT, or MRI of the neck, 18F-FDOPA PET/CT possibly with 18F-FDG PET/CT, and then targeted imaging depending on specific organ involvement (Figure 5). However, future studies that measure patient outcomes and cost-effectiveness are warranted.

In some patients, biological markers (CNT and CEA levels and doubling times) may not suffice for surveillance since they may not address the heterogeneity of response at different sites as therapy-resistant clones develop [61]. Given the availability of powerful radiological technologies, there is a need to perform comprehensive studies to evaluate correlations between biological markers and the location of the metastases in patients with advanced MTC in order to establish imaging protocols and diagnostic algorithms.

#### 3.2.1. Lymph Nodes

US of the neck is the most accurate modality for the detection of recurrent disease in the neck [27]. US features that should arouse suspicion about lymph node metastases include a rounded bulging shape, increased size, replaced fatty hilum, irregular margins, heterogeneous echotexture, calcifications, cystic areas, and vascularity throughout the lymph node instead of normal central hilar vessels on Doppler imaging [62]. US is less accurate for detecting central compartment disease than in the lateral compartments, as up to 80% of patients will have metastases to the central neck nodes. One of the most common pitfalls in cervical US is not to examine the lateral neck. If US suspects metastatic lymph nodes, then contrast-enhanced CT of neck and chest (for the evaluation of mediastinum) is a reasonable next step in imaging. Suspicious enhancing or abnormally appearing neck lymph nodes should undergo fine needle aspiration (FNA) with CTN assay of washout fluid. The reliable identification of mediastinal lymph node metastases under 1 cm is challenging, as mediastinal lymph nodes under 1 cm are considered normal on CT examination, and FNA of these nodules is not feasible. Therefore, subsequent 18F-FDOPA PET/CT may localize these small metastatic foci and provide guidance for surgeons.

#### 3.2.2. Imaging of Distant Metastases

**Figure 6:**
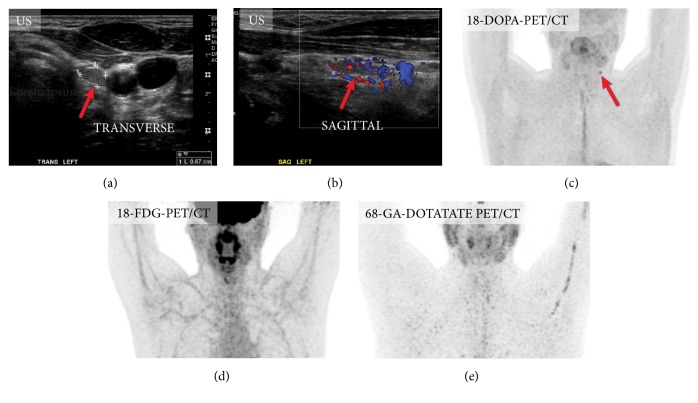
Mapping of the metastatic lymph nodes in medullary thyroid carcinoma. Asymptomatic patient after thyroidectomy for medullary thyroid carcinoma and elevated calcitonin level (54 pg/ml) was evaluated for possible neck lymph node metastases. Ultrasound of the neck showed several suspicious lymph nodes (not shown). One of the lymph nodes (b) was found to be positive on only 18F-FDOPA-PET/CT (c), while 18F-FDG-PET/CT and 68Ga-DOTATATE-PET/CT were negative.

**Figure 7:**
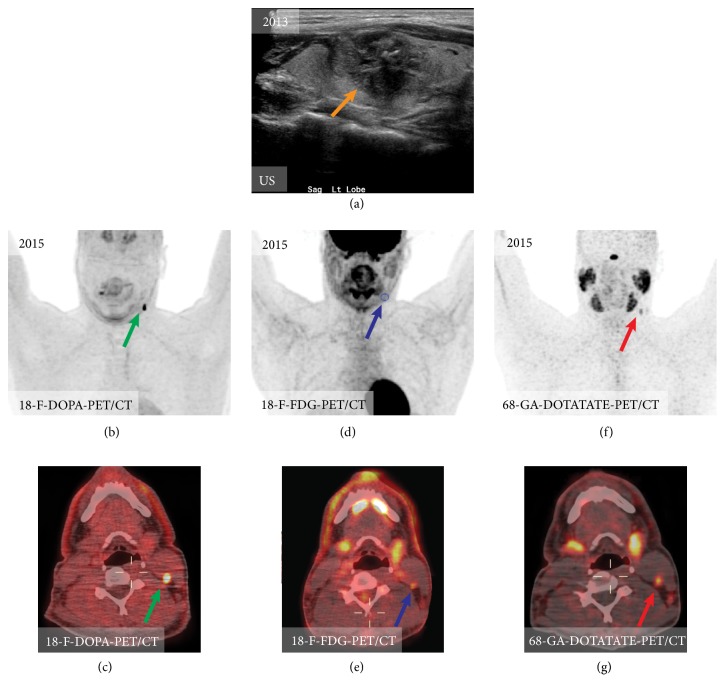
Mapping of the metastatic lymph nodes in medullary thyroid carcinoma. In 2015 patient underwent thyroidectomy for medullary carcinoma (a) and 2 years later presented with elevated calcitonin level (304 pg/ml). Ultrasound of the neck, CT of the neck, and MRI of the neck were all false negative (not shown); however, 18F-FDOPA-PET/CT (c), 18F-FDG-PET/CT, and 68Ga-DOTATATE-PET/CT were positive for a metastatic lymph node. Please note that patients with neck pathology should ideally be scanned with arms down (in this case the study was obtained with patient's arms up as it was a whole-body imaging for detection mediastinal metastases).

Currently, the further imaging for the detection of distant metastases is recommended when the postoperative serum CTN level is >150 pg/ml (551 pmol/l) [63]. Even though no step-by-step recommendations for patients with occult elevated CTN and CEA levels were suggested, the published studies have confirmed the superiority of 18F-FDOPA PET/CT over all other radiopharmaceuticals in the localization and detection of occult metastatic lesions [23, 29] (Figure 6). 18F-FDOPA PET/CT demonstrated the highest sensitivity in the detection of the metastatic lymph nodes compared to 18F-FDG and 68Ga-DOTATATE PET/CT [23]. 18F-FDOPA retention is a feature of high differentiation, while the accumulation of 18F-FDG reflects poor differentiation of the MTC cells [23]. For the imaging of recurrent disease, the two tracers act in a complementary manner to provide the maximum benefit from their combined sensitivity [23] (Figure 7). 18F-FDOPA still remains an 18-F-FDOPA is still not FDA approved diagnostic radiopharmaceutical for MTC in the USA and is approved for clinical use in some European countries. Unfortunately, not all institutions have 18F-FDOPA available; therefore, in many cases patients need to be referred to specialized centers in the US and Europe.

For distant metastases, nuclear medicine imaging with different radiopharmaceuticals is considered a type of “screening technique”: when the metastatic involvement of the particular organ is suspected or identified, a targeted evaluation of the organ with cross-sectional imaging is needed.

**Figure 8:**
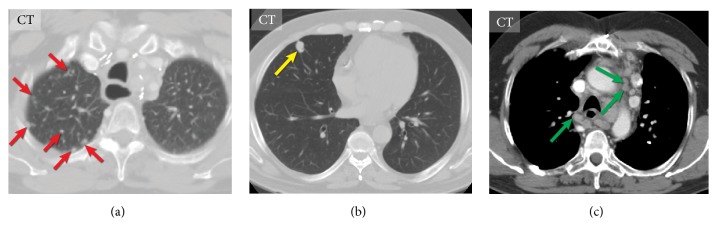
Computed tomography of the chest of different patients with metastatic medullary thyroid carcinoma. Disseminated pulmonary metastases (red arrows) (a). Solitary pulmonary metastases (yellow arrow) (b). Extensive metastatic mediastinal lymphadenopathy (green arrows) (c).

*(1) Lung Metastases*. Lung metastases occur in 33% of patients with MTC [27]. They are typically numerous and may be associated with mediastinal lymph node metastases [63]. Pulmonary metastases in MTC may exhibit a micronodular pattern or have a large, well-circumscribed, round macronodular appearance, also known as a “cannonball” pattern. Micronodular metastases have a nonspecific appearance, which may calcify and can be easily mistaken for granulomatous diseases, such as tuberculosis, histoplasmosis, or sarcoidosis [64, 65]. A lymphangitic spread of the tumor with amyloid deposition in peribronchovascular structures without alveolar involvement resulting in reticulonodular perihilar opacity on imaging was also reported [66]. Chest radiograph is a insensitive technique to diagnose lung metastases; about half of pulmonary lesions < 5 mm remain undetected [67]. CT is excellent at visualizing pulmonary metastases; they appear as well-circumscribed round soft tissue lesions, most often in the lower lungs. MR imaging is not commonly used for follow-up diagnosis or evaluations of pulmonary metastases. Pulmonary lesions larger than 5 mm may be readily identified on MR imaging; however, smaller nodules are detected with less sensitivity [68]. Therefore, MR imaging cannot replace CT for the diagnosis of pulmonary metastases. DWI was proven to be a useful technique for differentiation between malignant and benign pulmonary nodules or masses in oncology; however, it has been never tested on MTC patients [69] (Figure 8).

*(2) Liver Metastases*. Liver metastases occur in 25–30% of patients with MTC [70, 71] and 45% of patients with advanced MTC [27]. Clinically occult liver metastases are the leading cause of failure to achieve biochemical cure with surgery [72]. On imaging, liver metastases are often small, numerous, and disseminated throughout the parenchyma; they grow slowly, and patients remain asymptomatic for a long time [63]. In one study, the size of the largest liver lesion ranged from 25 to 98 mm (median, 38 mm) [73]. Liver metastases in MTC may have an initial miliary pattern, which makes identification difficult, and lesions may be missed [72]. Calcification of these metastases is seen in about one-third of cases [70]. Large, coarse, and irregular calcifications are more typical for MTC; however, small calcified metastases can also be seen. Extensive metastatic disease of the liver may lead to acute liver failure [74].

**Figure 9:**
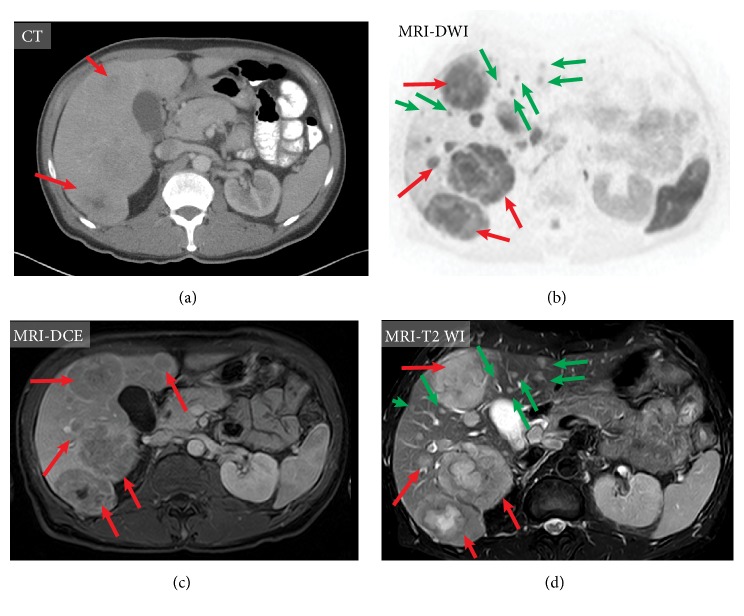
Imaging of hepatic metastases in medullary thyroid carcinoma of the same patient using contrast-enhanced computed tomography (CT) (a), magnetic resonance imaging (MRI), diffusion-weighted sequence (b); contrast-enhanced MRI, T1 weighted images (DCE)(c); MRI, T2 weighted image (d). MRI shows improved visualization of metastatic lesions compared to CT (red arrows) and can also detect very small metastases (green arrows), which may not be visible on CT.

Ultrasound, CT, MR imaging, and PET/CT can all be used to identify hepatic metastases. The goals of imaging are to identify the location of all metastatic tumors and to determine the feasibility of local resection or the application of other therapeutic or interventional modalities. The sensitivity of US to detect and characterize metastases remains low, ranging from 50 to 76% [75], and cannot be reliably recommended for screening or following up on patients with metastatic MTC. Four sonographic patterns of MTC hepatic metastases were described [76]; however, in era of MRI and functional imaging, it has little clinical application. The vast majority of MTC hepatic metastases were described as hyperechoic lesions and may be mistaken for hemangiomas [76, 77]. CT scans of the liver may not reveal the usual, nodular infiltration if there is diffuse intrasinusoidal spread [74]. The use of a multiphase contrast abdominal CT has proven useful in other NETs and may improve the detection of macroscopic MTC liver metastases [72]. MTC hepatic metastases are often hypervascular and have no specific radiological features on CT. Most metastases are revealed as hyperdense during hepatic arterial phase and hypodense or isodense masses on contrast-enhanced CT during the portal venous phase [78]. MRI is considered superior to CT for the detection and characterization of small liver lesions. The majority of metastases show a hypointense to isointense signal on T1-WI, and an isointense to hyperintense signal on T2-WI with enhancement findings similar to those on CT. The diagnostic accuracy of standard sequences with contrast enhancement and DWI technique is equal; therefore DWI appears to be an alternative option for standard MRI protocols—it is fast and easy to perform and seems to be especially valuable for imaging in detection of liver deposits, particularly during patients' clinical follow-up period. DWI sequences improve detection rates and possibly improve the characterization of small (5–10 mm) lesions in patients with NETs [79]. A single DWI scan could also be performed frequently, particularly in those patients who receive active therapy or have progressive cancer, with a potential need for changes in therapeutic management [79]. DCE MR imaging is the most sensitive imaging study for liver metastases [80]. It was found to be an effective tool in assessing and predicting the response to peptide receptor targeted radionuclide therapy in patients with NET liver metastases [81]; however, no similar studies on MTC have been performed. 18F-FDOPA is considered the best radiopharmaceutical to identify metastatic liver lesions [23]. The sensitivity of contrast-enhanced MR imaging retrospectively fused with 18F-FDG PET in the detection of liver non-MTC metastases is higher than that of 18F-FDG PET/CT [82]. Therefore, it is expected that the PET/MR imaging technique utilizing 18F-FDOPA tracer may be very helpful for detection and treatment monitoring in MTC (Figure 9).

*(3) Bone Metastases*. Bone metastases occur in 19–54% of patients with advanced MTC [27, 83]. Up to now, the largest series of 188 MTC patients with bone metastases demonstrated that MTC metastases are almost uniformly multifocal, with 77% of patients having at least 6 documented lesions, with the spine and pelvis being the most common locations of the lesions. About half (48%) of patients developed skeletal-related events, the majority of which affected the spine (58%), pelvis (17%), and extremities (11%) [83]. About 14% of bone metastases were diagnosed before evidence of nonosseous distant metastases, 24% had concomitant bone metastases identified at the time of distant metastases diagnosis, and 62% had bone metastases diagnosed after nonosseous distant metastases diagnosis [83]. The radiological appearance of MTC bone metastases is not specific. On CT, lesions may be osteolytic, osteoblastic, or mixed [80]. On MR imaging, they usually have low signal T1-WI and enhance after contrast administration, similar to other metastases. Bone scan of osteoblastic metastases usually demonstrates focal increased uptake of radioactivity, whereas lytic bone metastases may appear as photopenic areas unless they cause a pathologic fracture, which manifests itself with increased tracer uptake.

**Figure 10:**
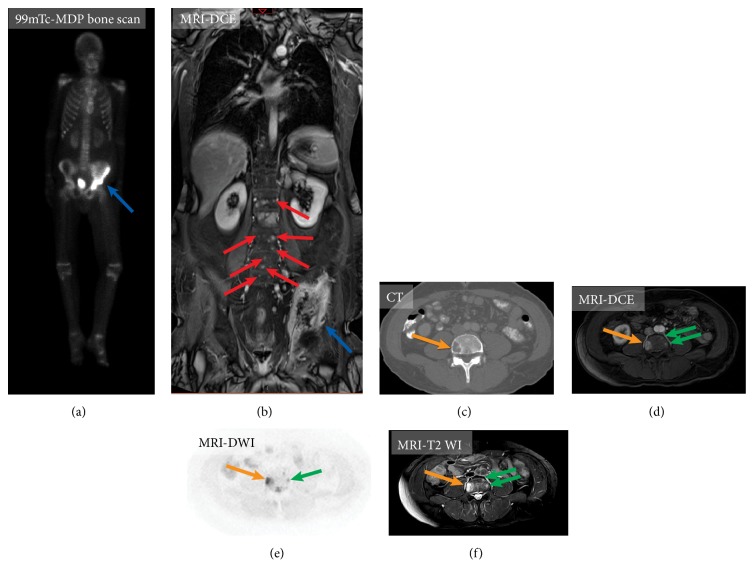
Imaging of bone metastases in medullary thyroid carcinoma on two different patients. Patient A (a-b). 99m-Tc-MDP bone scan (a) and whole-body contrast-enhanced magnetic resonance imaging (MRI), T1 weighted images (DCE) (b). MRI demonstrates improved visualization of metastatic lesions involving the left iliac bone (blue arrow) and clear visualization tiny bone metastases in the spine (red arrows). Patient B (c-f). Visualization of small bone metastases. Axial contrast-enhanced computed tomography (CT) at the level of L1 shows single lytic metastases in the right aspect of the vertebral body (orange arrow) (c). Axial contrast-enhanced MRI, T1 weighted images (DCE) (d), axial MRI diffusion-weighted sequence (e), and axial MRI, T2 weighted images (f) can detect additional small spinal metastases, which are not visualized on CT (green arrows).

Up to now, there is no clear answer to the question as to which modality is the most accurate for the identification of bone MTC metastases, as no recent studies evaluating the sensitivity and specificity of different imaging modalities in MTC bone metastases and the assessment of the response to the therapy have been performed. Two early studies showed contradictory results: Mirallié et al. (2005) demonstrated a higher sensitivity of MR imaging for detecting bone metastases compared with bone scintigraphy, while Giraudet et al. (2007) claimed a better sensitivity of bone scans [27, 84]. In another small study, 68Ga-DOTATATE PET/CT demonstrated a high sensitivity in detecting bone lesions and potentially it may be considered a substitute for a bone scan and MRI [33]. The European Organization for Research and Treatment of Cancer (EORTC) imaging group stresses the superiority of MR imaging over bone scintigraphy for the detection of all bone metastases and suggests the use of whole-body MRI and 18F-FDOPA PET/CT for the detection of MTC bone lesions [85] (Figure 10).

### 3.3. MTC in Patients with MEN2 Syndromes

**Figure 11:**
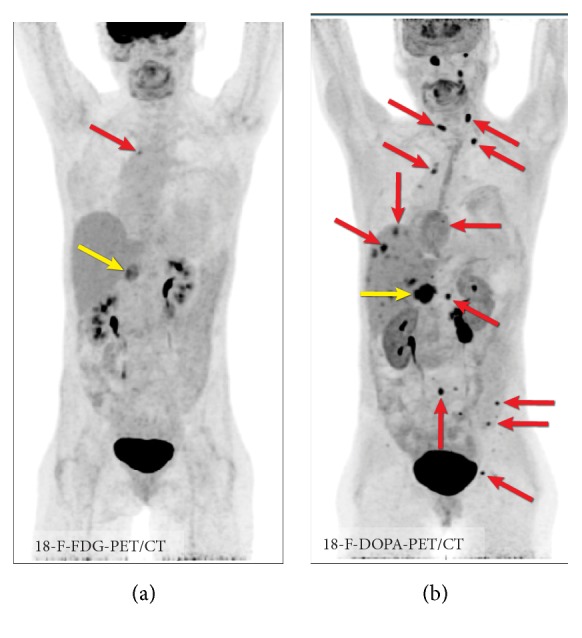
Imaging in MEN syndrome. 18F-FDG PET/CT (a) and 18F-FDOPA PET/CT (b) in MEN2B syndrome with metastatic pheochromocytoma (fractionated norepinephrine: 994 pg/ml, normal: 18-112 pg/ml; fractionated metanephrine: 1099 pg/ml, normal: 12-61 pg/ml) and metastatic medullary carcinoma (calcitonin: 5575, normal: < 8 pg/ml). In this case, the primary pheochromocytoma is seen in all images (yellow arrow); however, metastatic lesions are best visualized on 18F-FDOPA PET/CT (red arrows). Imaging cannot differentiate between medullary carcinoma metastases and metastases originating from pheochromocytoma.

Patients with MEN2 should be evaluated for pheochromocytoma before thyroid surgery for MTC. Therefore, serum calcium, phosphate, and parathyroid hormone levels, as well as catecholamine metabolites in plasma and urine, are part of the routine laboratory workup. Imaging cannot differentiate between medullary carcinoma metastases and metastases originating from pheochromocytoma (Figure 11).

## 4. Tumor Response Criteria in MTC

Accurate staging and evaluation of treatment response are critical for optimal treatment decisions in MTC. Response Evaluation Criteria in Solid Tumors (RECIST), the most commonly used universal system for the quantification of tumor response in oncologic imaging, has been used to assess the effects of MTC treatment medications in different studies and clinical trials [86–89]. However, it appears that the RECIST system is not ideal for MTC. Lesions smaller than 1 cm are considered “nonmeasurable,” and miliary MTC metastases cannot be assessed. Blastic bone metastases also remain “nonmeasurable” and, given the high rate of bone metastases in MTC, these lesions cannot be evaluated [85]. The majority of the studies devoted to MTC showed no correlation between RECIST response and biochemical markers—patients who responded to the therapy predominantly based on CNT level showed stable disease based on RECIST criteria despite apparent biochemical responses [13, 87, 88, 90–92]. Biological markers (CNT and CEA levels and doubling times) alone cannot be used as tumor response criteria, and they do not address the heterogeneity of response at different sites as therapy-resistant clones develop. A dedicated system for assessment of the MTC response is needed, which probably should include conventional and functional imaging including 18F-FDOPA and 18F-FDG PET/CT with relation to the biochemical markers.

## 5. Conclusions

Functional imaging, primarily PET/CT with 18F-FDOPA and 18F-FDG, plays a crucial role in the evaluation and management of MTC and has proven to be an efficient tool for the detection of metastases in patients with elevated CTN levels. Future research is needed to evaluate the role of advanced imaging techniques such as DWI, DCE, and hybrid PET/MR for MTC and to clarify the CTN cutoff for the follow-up taking into account the recent progress in imaging techniques. It can be suggested that any patient's elevated postoperative CTN should be considered as metastatic disease of unknown location and should prompt comprehensive radiological evaluation. These patients may benefit from 18F-FDOPA PET/CT with following targeted imaging depending on specific organ involvement.

Modern imaging techniques can provide an accurate assessment of a response to therapies and facilitate the translation of basic science into clinical application in clinical trials. Although a number of new imaging modalities evolved over the last decades and are effectively used in different oncological applications, the clinical and research use of these novel techniques are still limited in the comprehensive evaluation of MTC, and new studies focusing on MRI techniques are needed. It is important to develop a dedicated system for the assessment of the MTC response, which probably should include conventional and molecular imaging including 18F-FDOPA and 18F-FDG PET with relation to the biochemical markers, as it appears that the RECIST system is not ideal for MTC.
